# Training practices of Japanese elite team and combat sport athletes during the COVID-19 pandemic: an interview study with support staff

**DOI:** 10.3389/fspor.2025.1557774

**Published:** 2025-09-04

**Authors:** Daichi Yamashita, Kazuya Yamazaki, Takaki Yamagishi, Hana Tsubokura, Jad Adrian Washif, Iñigo Mujika

**Affiliations:** ^1^ Department of Sports Sciences, Japan Institute of Sports Sciences, Tokyo, Japan; ^2^ Department of Sports Medicine, Japan Institute of Sports Sciences, Tokyo, Japan; ^3^ Sports Performance Division, National Sports Institute of Malaysia, Kuala Lumpur, Malaysia; ^4^ Department of Physiology, Faculty of Medicine and Nursing, University of the Basque Country UPV/EHU, Leioa, Spain; ^5^ Exercise Science Laboratory, School of Kinesiology, Faculty of Medicine, Universidad Finis Terrae, Santiago, Chile

**Keywords:** detraining, self-isolation, coronavirus, training restriction, online training, strength and conditioning

## Abstract

**Background:**

The coronavirus disease 2019 (COVID-19) pandemic had a profound impact on the world of sports, particularly elite athletes. This study aimed to investigate how elite athletes adapted their training during the pandemic through semi-structured interviews with performance support staff from national (NT) and professional (PT) teams.

**Methods:**

Thirteen Japanese support staff (NT = 9, PT = 4) provided insights into the challenges of maintaining training duration, intensity, and quality, as well as strategies adopted to navigate specific phases, including “during” and “after” the nationwide State of Emergency and “during” and “following” two-week quarantines, through semi-structured interviews.

**Results:**

During the nationwide State of Emergency and subsequent quarantines, most NT and PT support staff reported that athletes had limited access to conventional training equipment and facilities, often relying on makeshift alternatives. When these options were insufficient, the support staff modified training content based on equipment availability. Moreover, many athletes voluntarily refrained from outdoor workouts to avoid negative public attention, despite no legal ban on outdoor training. Most NT and PT support staff relied on online platforms for group or personal sessions to help maintain fitness and motivation, although these methods were considered inadequate for achieving optimal outcomes. Upon returning from quarantine, support staff implemented phased return-to-play programs. For some athletes, the cancellation of competitions presented an opportunity to focus on thorough rehabilitation for chronic injuries. Furthermore, social isolation and uncertainty increased mental health concerns among athletes, emphasizing the need for greater psychological support. Elite athletes faced challenges, including limited indoor space, inadequate equipment, and lack of “domestic rivals” to optimize training performance.

**Conclusion:**

Overall, the pandemic experience emphasized the importance of robust, adaptable athlete-support systems capable of modifying training under constrained conditions. These findings highlight the value of well-defined return-to-play approaches, better communication among support staff, and holistic support strategies (well-being, etc.) to enhance resilience during future disruptions.

## 1 Introduction

The coronavirus disease 2019 (COVID-19) pandemic had a profound impact on the world of sports. Global lockdowns and subsequent restrictions in the first half of 2020 severely disrupted athletic activities, leading to facility closures, travel limitations, and social distancing protocols among other measures. These restrictions varied based on governmental and local regulations. Athletes faced considerable challenges, including the cancellation or postponement of major sporting events, such as the Tokyo 2020 Summer Olympics and various national and international competitions.

In response to these disruptions, many athletes personalized their training at home to maintain or increase their fitness and performance (1). In a large global cohort, Washif et al. (2) reported substantial reductions in key training variables (e.g., frequency, duration, intensity, type) during lockdowns. Notably, elite athletes engaged in more intensive and high-volume daily training than lower-level athletes, and a higher proportion of them reported being able to maintain their training (3). They coped relatively better with the training restrictions due to the greater availability of training equipment and support staff (2, 4), although these resources were limited in scope (1). However, the cancellation of international competitions due to travel restrictions further reduced opportunities for athletes to practice and compete against their rivals. Unlike recreational or lower-level athletes, whose involvement in sports activities is related to leisure and non-career pursuits, elite athletes [i.e., national team (NT) and professional team (PT) athletes] train vigorously on a full-time basis as part of their career objectives, striving to earn a living and win major events such as the Olympic Games.

Elite athletes, who typically depend on support staff for their training (5), faced considerable challenges during the lockdowns (1), with only half continuing to receive guidance from coaching staff (2). Lockdown training often required modifications to comply with restrictive policies, such as home-based training, and further constrained by resource availability (e.g., using household items) (1). This lockdown-induced training adaptation yielded variable outcomes, including improvements in certain fitness parameters, possibly due to a shift in focus towards previously neglected physical components (1, 6). In Japan, the nationwide State of Emergency brought about various restrictions, similar to other countries. These restrictions were either voluntary (7) or legally mandated under the Infectious Diseases Control Law (8, 9) and the Quarantine Act for Overseas Entry (9) (summarized in Table 1). For high-performance sports, these circumstances are particularly significant: (a) Japan was the host of the Tokyo 2020 Olympics (held in 2021); (b) there was substantial preparation by Japanese athletes and support staff for the Olympics, and its prominent Professional leagues (i.e., baseball, soccer, and basketball). Japan's third-place in gold medals at the 2020 Olympics may reflect its success in navigating the pandemic challenges. Nevertheless, training adaptations and strategies of Japanese athletes and support staff under these circumstances during the COVID-19 pandemic are virtually unknown, and have not been emphasized in previous studies regarding different national contexts (11–14).

**Table 1:** COVID-19 control measures and restrictions in Japan across different periods.

Items	During the State of Emergency	Post-State of Emergency period	Localized State of Emergency period	Post-State of Emergency
Period	April 7 (7 prefectures) or April 16 (all 47 prefectures), 2020–May 25, 2020	May 26, 2020–May 7, 2023	Intermittent by prefecture	May 8, 2023
Medical classification	Equivalent to Class 2 (mandatory measures allowed)	Equivalent to Class 2, gradually shifting to recommendations	Equivalent to Class 2, stricter measures reinstated when necessary	Class 5 (no mandatory measures; personal responsibility)
Quarantine (infected/close contacts)	Mandatory isolation (home or designated facilities)	Isolation for infected individuals; recommendations for close contacts	Varied by prefecture	Voluntary quarantine
Quarantine (overseas entry)	14-day mandatory isolation in designated facilities	Quarantine continued but gradually relaxed; periods reduced; exemptions for vaccinated travelers	Varied based on variants and infection rates	All border measures lifted
Outdoor activity restrictions	Strong requests to avoid non-essential outings; sports facilities closed	Gradual relaxation; reopening with infection controls	Stricter measures in high-risk areas; local restrictions applied	All restrictions removed; normal facility operations; infection control recommended

This is based on information from Kitahara et al. (8) and Ministry of Health, Labour and Welfare (9). ‘Class’ refers to classification under Japan's Infectious Diseases Control Law. During the Tokyo 2020 Olympics and Paralympics, an “Athlete Track” was introduced as a special measure approved by the Japanese government. This measure allowed athletes entering Japan to be exempt from the standard 14-day self-isolation required for international arrivals from qualifying competitions and pre-camp events (10).

Previous studies have conducted questionnaire surveys of athletes at different competitive levels during the COVID-19 pandemic, revealing substantial limitations to continue with regular training (2, 13, 15). However, these questionnaire surveys for athletes did not detail the specific restrictions faced (e.g., noted above) and how the support staff planned and implemented training programs to help athletes navigate the restrictions. Most, if not all, previous studies have primarily focused on the overall pre- and post-lockdown effects (2, 13, 15, 16), without the nuanced impact of multiple, evolving restrictions. Thus, we conducted interviews with support staff to enable a deeper exploration of context-specific details, with nuanced information about strategies and adaptations used to support athletes in training during different restriction conditions. Therefore, this study aimed to explore the training restrictions and practices of elite Japanese athletes during the COVID-19 pandemic through semi-structured interviews with NT and PT support staff. We focused on the support staff involved in team sports and combat sports training because they require diverse physical demands and typically involve multiple individuals training together, and they faced particularly severe restrictions during the pandemic (3). The findings will enhance our understanding of training management during major disruptions, such as pandemics, geopolitical or religious restrictions, adverse weather and climatic conditions, or local government impositions, where athletes must adapt to limited resources, confined spaces, and the absence of traditional team and support environments (1).

## 2 Methods

### 2.1 Participants

Thirteen Japanese support staff (12 men and 1 woman; age: 47 ± 8 years) who were either working for NT (*n* = 9) or PT (*n* = 4) participated in this study. PT support staff belonged to one of three team sports: baseball (Nippon Professional Baseball League; NPB League), soccer [Japan Professional Football League; J.League (first division)], and basketball [Japan Professional Basketball League; B.League (first division)] (Table 2). To prevent the potential identification of NT staff interviewees, each sport was classified into a category as in Washif et al. (3) (Table 2). Participants provided written informed consent to participate in the study, which was conducted in accordance with the Declaration of Helsinki and was approved by the Japan Institute of Sports Sciences Ethics Committee (No. 2021-056).

**Table 2:** Response and position summary of national team (NT) and professional team (PT) support staff (interviewees).

Team	Sport classification	Sport environment	Sport gender	Interviewee gender	Interviewee position
NT	Combat	Indoor	Men	Man	S&C coach
		Indoor	Men	Man	Therapist
		Indoor	Women	Woman	S&C coach
		Indoor	Men/Women	Man	S&C coach
		Indoor	Men/Women	Man	Therapist
	Team	Outdoor	Men	Man	S&C coach
		Outdoor	Men	Man	S&C coach
		Indoor	Men	Man	Assistant coach
PT	Team	Outdoor	Men	Man	S&C coach
		Outdoor	Men	Man	S&C coach
		Indoor	Men	Man	S&C coach
		Indoor	Men	Man	S&C coach
		Indoor	Men	Man	High performance director

S&C, strength and conditioning.

### 2.2 Procedures

Interviews were conducted either in person or via online meeting platforms between January and April 2022. The duration of each interview was approximately 47 ± 8 min. The session began with a quantitative question, where interviewees were asked to evaluate or report their training duration, intensity, and quality during the nationwide State of Emergency, using their regular training benchmarks set at (equivalent to) 100% as reference. This assessment was applied to the following specific training categories: cardiorespiratory, strength and power, speed and agility, mobility, and sports-specific skills. Following the quantitative evaluation, the interviews transitioned to semi-structured inquiries based on pre-defined core themes/questions, allowing for open-ended responses. NT support staff responsible for individual athletes, rather than the entire team, were asked to reflect only the circumstances of those individuals.

The research team formulated these core questions with the following focuses:

1. Challenges encountered in maintaining training duration, intensity, and quality, and the strategies adopted to navigate those challenges during specific times such as “during the nationwide State of Emergency”, “after the nationwide State of Emergency”, “during the two-week quarantine”, “following the two-week quarantine”, and “throughout the COVID-19 pandemic period”.
2. Insights into changes in athletes' physical fitness following “the nationwide State of Emergency” and “two-week quarantine”. This encompassed the duration required for athletes to return to their baseline fitness and the incidence of injuries, along with their potential causes.

Finally, we asked a broad question to the support staff: “Reflecting on the COVID-19 pandemic, please freely share the tangible outcomes, challenges that became evident, and any recommendations or insights for the future”.

The interviewees' responses included not only their own actions but also observation of their athletes and actions taken by other team staff. In addition to the core question items, we addressed topics brought up by the interviewees, provided they were aligned with the research objectives. The interview was concluded when all the pre-prepared core question items were covered and the interviewee's narrative concluded smoothly. All interviews were recorded with interviewees' agreement. Verbatim transcripts were then generated and converted into text. Subsequently, a qualitative content analysis was conducted, following the methods of Elo and Kyngas (17). The transcripts were carefully read, and descriptions were extracted and coded based on their semantic content. Subcategories and categories were derived by comparing similarities and differences while raising the level of abstraction. Subsequently, these categories were integrated and themed. The analysis was conducted collaboratively by multiple researchers, including an experienced researcher of content analysis. Regular discussions (i.e., category generation) were held to ensure reliability and validity. In addition, the authors were involved in the entire analytical process, and discussions persisted until a consensus on the interpretation of the data was reached.

### 2.3 Data collection and analysis

Although a total of 13 support staff were interviewed, four participants did not provide responses for all periods or questions owing to variations in their roles and timelines. Specifically, one NT support staff member (from a combat sport) had not started working with the team during the nationwide State of Emergency and, therefore, could not comment on that period. Additionally, two NT and one PT support staff were not involved in team management or activities; thus, they did not participate in the quantitative survey, but contributed to the semi-structured interviews.

The semi-structured interviews were not standardized across all participants because the questions asked depended on each staff member's role and experience. Consequently, the frequency of responses was not quantified. Instead, we highlighted the key insights that emerged from the interviews, irrespective of whether they were mentioned by multiple or single participants. This approach ensures that unique and potentially important strategies or challenges were fully documented. From the 241 extracted descriptions, only the primary categories and their key representative examples (195 descriptions) are included in the main table, while the remaining descriptions (46 descriptions) are summarized in the Supplemental Materials (Supplementary Tables S1–S6).

For the quantitative part of the interview, interviewees provided specific numbers regarding the training duration, intensity, and quality during the State of Emergency. However, some of the answers provided had a range (e.g., 5%–10%). Given that some responses were presented as ranges, we opted to categorize them at 25% intervals to facilitate the analysis.

Regarding two-week quarantine periods, we classified them into four scenarios: (1) quarantine due to being infected with COVID-19; (2) quarantine due to close contact and other factors; (3) group isolation (including “Athlete Track”), a special measure for the Tokyo 2020 Olympics and Paralympics approved by the Japanese government, allowing athletes entering Japan to be exempt from the standard 14-day self-isolation required for international arrivals from qualifying competitions and pre-camp events (10); and (4) quarantine from international competitions. Athletes who tested positive were isolated according to government guidelines.

## 3 Results

One notable impact of the nationwide State of Emergency on training was the limitation in sports-specific skill training for athletes (Table 3). All five NT support staff reported that the duration, intensity, and quality of sports-specific training decreased to below 50% of the normal levels. Among the four PT, one reported maintaining over 75% of their usual sport-specific training in terms of duration, intensity, and quality, while others did not manage to maintain it to the same extent. In contrast, cardiorespiratory and mobility training durations remained close or exceeded the normal levels in most NT and PT, with similar intensity and quality. For strength and power training, six teams (3 NT and 3 PT) achieved over 50% of their usual duration, but only four maintained over 50% of the typical intensity and quality. For speed and agility training, only three (1 NT and 2 PT) support staff secured over 50% of the usual duration and intensity.

**Table 3:** Training duration, intensity, and quality during the State of Emergency.

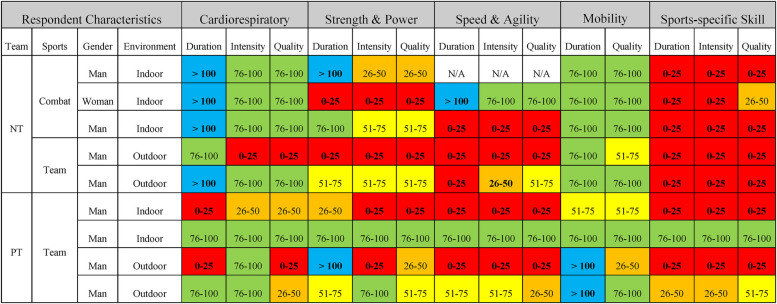

Numbers are standardized to a baseline of 100 under regular conditions. Data are categorized into quartiles to address ambiguity in specific metrics provided by the support staff. NT, national team; PT, professional team; N/A, not applicable; Blue, Over 100%; Green, 76%–100%; Yellow, 51%–75%; Orange, 26%–50%; Red: 0%–25%.

During the nationwide State of Emergency, limitations on facility access greatly impacted training routines for both indoor and outdoor sports (Table 4). Nonetheless, support staff and athletes implemented various strategies to address these challenges. Due to information uncertainty and public sentiment, four teams (2 NT and 2 PT) voluntarily refrained from outdoor training. Support staff often provided guidance on recommended training (4 NT and 2 PT) and/or facilitated online training sessions (3 NT and 3 PT). The majority of support staff mentioned that athletes prepared small training equipment (e.g., dumbbells, training tubes) for their workouts (4 NT, 4 PT), and fewer support staff (2 NT, 2 PT) reported that their athletes had access to larger equipment (e.g., power racks, cycle ergometers). Additionally, training programs and their management were often independently determined by the athletes or by non-NT/PT support staff, such as personal support staff from their home teams (5 NT and 2 PT). Due to their body size and dynamic, powerful movements, athletes faced limitations in training spaces (1 PT) and found commercial equipment inadequate for their training needs (1 PT). Mental health challenges emerged among athletes, partly due to the inability to train as usual (2 NT and 1 PT).

**Table 4:** Support staff [8 national team (NT) and 4 professional team (PT)] response in interviews regarding the situation of athletes and support staff during the nationwide State of Emergency (39 descriptions).

Categories	Subcategories	Descriptions	Total (NT, PT)
Level of restrictions	Facility closure or restriction	Athletes had no access to their main training centers.	10 (7, 3)
		Athletes were able to use their teams’ facilities within a limited amount of time.	3 (2, 1)
		Athletes living in team dormitories could use the training facilities there.	2 (1, 1)
		Athletes conducted practice or training sessions without coaching staff.	1 (0, 1)
		Support staff and athletes were able to train at their teams’ facilities but limited amount of sport-specific training with their teammates.	1 (1, 0)
	Self-isolation	Athletes had no idea what to do due to a lack of information on COVID-19.	2 (2, 0)
		The health of athletes and of those around them was prioritized.	2 (2, 0)
		Due to “virus vigilantes”, athletes could only train at home.	4 (2, 2)
	Others	Since national teams are typically organized prior to training camps or specific games, support staff could not continuously supervise the athletes.	1 (1, N)
Actions of athletes/staff	Communication	Support staff regularly contacted athletes for general conversation.	2 (2, 0)
		Support staff had regular online meetings with athletes regarding their sport-specific issues.	2 (2, 0)
	Provision of information	Support staff provided training programs to athletes.	6 (4, 2)
		Support staff provided athletes with information on available training equipment.	3 (1, 2)
	Training	Training programs and their management were decided either by the athletes themselves or by their staff, who were not NT/PT support staff.	7 (5, 2)
		Support staff checked physical issues online and advised accordingly.	7 (6, 1)
		Support staff monitored their training progress.	2 (2, 0)
		Support staff prescribed high volume (repetition) training.	1 (1, 0)
		Support staff prescribed high-velocity training.	1 (1, 0)
		Support staff organized small-group training within a limited amount of time to minimize the risk of infection.	2 (0, 2)
		Support staff asked instructors to deliver online yoga sessions.	3 (2, 1)
		Support staff supervised athletes’ practice and/or training online.	1 (0, 1)
		Support staff delivered online training sessions.	6 (3, 3)
		Support staff prescribed training that had not been frequently done before COVID-19.	4 (3, 1)
		Athletes purchased small training equipment (e.g., dumbbells, training tubes, pull-up bars).	8 (4, 4)
		Athletes purchased large training equipment (e.g., power rack and cycle ergometer).	4 (2, 2)
		Athletes sought places to work out (e.g., park).	2 (1, 1)
		Athletes performed circuit training by themselves.	2 (1, 1)
		Athletes did individual training sessions with sport-specific equipment.	4 (3, 1)
	Recovery	Athletes had their injuries treated (including surgeries).	3 (3, 0)
	Mental health	Support staff kept athletes motivated by holding online training sessions with athletes.	1 (1, 0)
	Communication	Support staff had difficulty keeping track of athletes’ conditions online.	4 (4, 0)
Challenges for athletes/staff	Training	Athletes had difficulty finding places (e.g., hills, stairs) where they could sprint outdoors.	2 (2, 0)
		Athletes had difficulty obtaining large training equipment for use.	1 (1, 0)
		Athletes were unable to perform dynamic training effectively indoors, constrained by their body size and the need for extensive movement space.	1 (0, 1)
		Athletes had difficulty adjusting intensity with commercial training equipment (e.g., rubber band) due to their physical characteristics.	1 (0, 1)
	Mental health	Athletes lost their motivation due to lack of training partners.	1 (1, 0)
		Athletes felt anxious and lost their sense of purpose to train when the State of Emergency was extended.	1 (1, 0)
		Athletes felt anxious about not being able to train sufficiently.	1 (0, 1)
		Athletes had difficulty maintaining motivation for online training compared to regular training.	1 (0, 1)

N, not applicable. Other minor descriptions are provided in Supplementary Table S1.

Even after the end of the nationwide State of Emergency, training restrictions persisted (Table 5). The support staff divided the athletes into small groups for training sessions (3 NT and 1 PT) and provided information about retraining (2 NT). The majority of support staff (4 NT and 3 PT) reported a gradual increase in training load. Regarding athletes' physical fitness, only one PT support staff reported no change, while all other support staff reported some form of detraining (Table 6). The majority of support staff observed a decline in strength and power, indicating that approximately two months were required to return to pre-pandemic levels (2 NT and 2 PT). Regarding endurance, two to four months were necessary for athletes to regain their normal levels (5 NT and 3 PT). None of the support staff reported a need for more than two months to recover body composition. Remarkably, four support staff (3 NT, 1 PT) reported that more than six months were required for athletes to recover their sports skills or performance. Regarding unusual injuries or disorders, five support staff (4 NT and 1 PT) reported none, but there were reports of muscle cramps (1 NT and 1 PT) and joint pain (2 NT and 1 PT).

**Table 5:** Support staff responses [9 national team (NT) and 4 professional team (PT)] in interviews regarding the situation of athletes and support staff after the nationwide State of Emergency (11 descriptions).

Categories	Subcategories	Descriptions	Total (NT, PT)
Level of restrictions	Social distancing and travel restrictions	Teams had few practice games with other teams in preseason.	1 (0, 1)
		Most sport-specific practice was done in small groups, and the number of athletes involved was limited.	2 (1, 1)
		Support staff were not available for a month after the State of Emergency.	1 (1, 0)
Actions of athletes/staff	Communication	Support staff had regular online meetings with athletes regarding their training.	1 (1, 0)
	Providing information	Support staff provided athletes with recommendations for retraining.	2 (2, 0)
	Training	Training program (and its operation) was dependent on each athlete.	1 (1, 0)
		Support staff gradually increased training load (intensity and volume).	7 (4, 3)
		Support staff divided the athletes into small groups and held training sessions or camps.	4 (3, 1)
	Recovery	Support staff instructed athletes to conscientiously take care of their connective tissues (muscles and tendons).	1 (0, 1)
	Others	Athletes’ eating habits actually improved due to the restrictions on outings at camp or competition venues.	1 (0, 1)
Challenges for athletes/staff	Training	Most of support staff in the team were impatient.	1 (0, 1)

Other minor descriptions are provided in Supplementary Table S2.

**Table 6:** Support staff responses [9 national team (NT) and 4 professional team (PT)] regarding changes in performance elements and time taken to return to baseline after the nationwide State of Emergency (31 descriptions).

Categories	Subcategories	Descriptions	Total (NT, PT)
Change in performance	General fitness	No change, as they were able to train.	1 (0, 1)
		Within 4 weeks.	1 (0, 1)
		4–8 weeks.	1 (1, 0)
	Strength/power	No/slight change.	1 (1, 0)
		Within 1 month.	2 (2, 0)
		2 months.	4 (2, 2)
	Speed/agility	4 months.	1 (0, 1)
	Endurance	2 months.	4 (3, 1)
		3 months.	1 (0, 1)
		4 months.	3 (2, 1)
	Body composition	For men, no change.	2 (2, 0)
		For men, body weight has not changed, but their body composition seemed to have worsened.	1 (1, 0)
		For men, 1–2 month.	1 (0, 1)
		For women, body composition had worsened (no mention of the duration).	2 (2, 0)
		For women, 1 month.	1 (1, 0)
	Perception and action	3–4 months.	1 (1, 0)
	Sports skill/Performance	1–2 months.	3 (2, 1)
		3 months.	1 (1, 0)
		More than 6 months.	4 (3, 1)
		Never returned without international competition.	1 (1, 0)
Unusual injury	None	No noticeable issues.	5 (4, 1)
	Muscle	Many chronic lower limb injuries.	1 (0, 1)
	Tissue other than muscle	Feet, arms, and fingers became more prone to cramping.	2 (1, 1)
		Fingertip joints became painful, and the skin weakened, making it easier for wounds to form.	2 (2, 0)
		Problems with joints (bones, ligaments, and connective tissues).	1 (0, 1)
	Cause	Increasing the amount of unfamiliar training.	2 (1, 1)
	Recovery	Inadequate recovery led to fatigue and muscle pain.	2 (1, 1)
Others	Preparation for the resumption of official matches.	Competitive performance was not regained in official matches three months after resumption.	1 (1, 0)
		Athletes regained their physical fitness levels in time for the official matches 3 months after resumption.	2 (0, 2)
		Athletes who joined late did not make it in time for the official matches three months after resuming.	2 (0, 2)
		Almost no one was able to return to their original performance in official matches that resumed within two months after the restart.	2 (0, 2)

For quarantine due to close contact and other factors, many support staff communicated with athletes online, provided training program information (2 NT and 1 PT), and conducted online training sessions (1 NT and 4 PT) (Table 7). Athletes trained using large equipment or small equipment, as primarily reported by PT support staff. In cases of quarantine following international competitions, only NT encountered this scenario. Five support staff reported that, since there was usually an off period after international competitions, athletes initially prioritized rest, as was typical during the pre-pandemic period. When group isolation was possible, such as with the “Athlete Track”, staff members accompanied the teams and conducted training sessions.

**Table 7:** Support staff responses [9 national team (NT) and 4 professional team (PT)] in interviews regarding the situation of athletes and support staff during a two-week quarantine (32 descriptions).

Categories	Subcategories	Descriptions	Total (NT, PT)
In case of being infected with COVID-19			
Actions of athletes/staff		Isolation measures were implemented based on government rules.	1 (1, 0)
In case of being quarantined due to close contact and other factors			
Actions of athletes/staff	Communication	Support staff provided athletes with training programs which were doable during quarantine.	3 (2, 1)
		Support staff and athletes reviewed sport-specific skills together online.	1 (1, 0)
		Support staff secured a time for online communication with athletes.	2 (1, 1)
	Mental health	Support staff sought to reduce athletes’ stress by discussing post-quarantine training plans in advance.	1 (1, 0)
	Training	Support staff created videos of training examples and provided them to athletes.	3 (2, 1)
		Support staff made schedules for returning to a team after quarantine.	1 (0, 1)
		Support staff delivered online training sessions.	5 (1, 4)
		Athletes trained using large equipment (e.g., cycle and rowing ergometers, benches, racks, hex bars).	7 (2, 5)
		Athletes trained using small equipment (e.g., kettlebells, weight bags, dumbbells, training tubes, medicine balls, skipping ropes, yoga mats).	5 (1, 4)
		Support staff prescribed high-volume training because high-intensity training was difficult to perform.	2 (0, 2)
		Support staff prescribed high-intensity isometric training.	1 (0, 1)
		Support staff prescribed high-intensity eccentric training.	2 (0, 2)
		Support staff developed individualized training plans using readily available resources, adapting them to athletes’ specific needs during quarantine.	2 (2, 0)
		Athletes independently searched for training video online and trained accordingly.	1 (1, 0)
		Athletes trained outside when a situation allowed.	4 (3, 1)
		Athletes performed bodyweight exercises.	3 (2, 1)
		Support staff designed training programs whose effects would last throughout the quarantine period.	2 (1, 1)
	Others	Support staff provided nutritional guidance to the athletes.	1 (0, 1)
Challenges for athletes/staff	Training	There were many restrictions on conducting training for athletes, and support staff set their primary goal as simply staying healthy.	1 (1, 0)
		The hotel spaces provided to athletes were small, limiting the types of training they could perform.	1 (1, 0)
	Mental health	The stress of quarantine was overwhelming for athletes.	1 (1, 0)
	Others	The provided hotel meals were nutritionally inadequate for athletes.	1 (0, 1)
		Athletes’ lifestyle habits became disrupted.	1 (1, 0)
In case of quarantine from international competitions			
Actions of athletes/staff	Training	Support staff reserved large hotel rooms for athletes.	1 (1, N)
		Athletes trained at low intensity.	1 (1, N)
		Athletes initially treated the off period following international matches as a time for recovery (as was typical during the pre-pandemic period), and subsequently transitioned to individually managed training.	5 (5, N)
Challenges for athletes/staff	Training	The stress experienced by athletes exceeded the expectations of support staff, prompting a mid-course adjustment to reduce training intensity.	1 (1, N)
		Support staff could not arrange suitable training rooms because the government designated the hotel.	1 (1, N)
In case of group isolation (including “Athlete track”)			
Actions of athletes/staff	Training	Support staff accompanied the team for camp-style training sessions.	4 (3, 1)
		Non-quarantined staff transported training equipment to facilities where quarantined athletes were staying.	1 (0, 1)
	Recovery	Medical staff brought in and used physical therapy equipment.	1 (1, 0)

N, not applicable. Other minor descriptions are provided in Supplementary Table S3.

After two weeks of isolation, the majority of support staff (5 NT and 3 PT) reported a gradual increase in training load (Table 8). Among them, four (2 NT and 2 PT) support staff adopted strategies to partially modify training content while rejoining other athletes. In fact, few support staff reported maintaining fitness levels during the two-week isolation, with most suggesting a recovery period of 1–4 weeks (Table 9). For athletes who tested positive, the common recovery time reported was 2–4 weeks. While specific injuries upon resuming training were not commonly prevalent, there were some reports of lower limb muscle pain.

**Table 8:** Support staff responses [9 national team (NT) and 4 professional team (PT)] in interviews regarding the situation of athletes and support staff after a two-week quarantine (5 descriptions).

Categories	Subcategories	Descriptions	Total (NT, PT)
Actions of athletes/staff	Training	Support staff gradually increased the training load for athletes step by step.	8 (5, 3)
		Support staff adjusted strategies to partially modify training content while athletes were rejoining their teammates.	4 (2, 2)
		Support staff controlled the training load by moderating players’ eagerness.	1 (0, 1)
		Support staff prescribed recreational activities.	1 (1, 0)
	Recovery	Support staff instructed players to take care of their connective tissues.	1 (0, 1)

Other minor descriptions are provided in Supplementary Table S4.

**Table 9:** Support staff responses [9 national team (NT) and 4 professional team (PT)] in interviews regarding changes in performance elements and time taken to return to baseline after a two-week quarantine (25 descriptions).

Categories	Subcategories	Descriptions	Total (NT, PT)
Change in performance	General fitness	More than 2 weeks for being infected with COVID-19.	1 (1, 0)
		After international matches involving quarantine, including the competition period, they were essentially unable to train for a month, so they needed 3–4 weeks to recover.	1 (1, 0)
		No change for those who were in a close contact because they could train from home.	1 (0, 1)
		1–2 weeks.	4 (3, 1)
		1–4 weeks.	1 (0, 1)
		2–4 weeks.	1 (1, 0)
		4 weeks.	1 (1, 0)
		No change if the quarantine period was 5–7 days, but there was a decline if it was longer than that.	1 (1, 0)
		1 week for fielders but 2–3 weeks for pitchers in baseball.	1 (0, 1)
		If it was 5–7 days, they had the impression that they could recover in 2–3 days afterwards.	1 (1, 0)
	Strength/power	No change.	1 (1, 0)
		1–2 weeks.	1 (1, 0)
	Speed/agility	1–2 weeks.	1 (1, 0)
	Endurance	Slightly decreased.	2 (2, 0)
		Recovery of the respiratory and circulatory systems was difficult.	1 (1, 0)
		Maintained if home training is possible.	1 (0, 1)
	Body composition	2 months to regain their body weight.	1 (0, 1)
		1 month for being infected with COVID-19.	1 (0, 1)
	Sports skill/Performance	1 week.	1 (0, 1)
	Others	Some athletes who had respiratory aftereffects for about a month after being infected with COVID-19.	1 (1, 0)
Unusual injury	None	No change.	5 (4, 1)
	Cause	Support staff wanted to start early and athletes were eager to get going, which led to injuries due to impatience.	1 (0, 1)
		The increased body weight of the athletes put greater strain on their bodies, causing pain.	1 (0, 1)
	Muscle	Athletes experienced pain in their lower limb muscles.	3 (1, 2)
	Recovery	Athletes’ recovery couldn't catch up, leading to fatigue and muscle pain.	1 (1, 0)

Finally, Table 10 provides a summary of overall responses, while Table 11 outlines achievements and challenges specific to the nationwide State of Emergency. Many support staff struggled to balance in-person training with the COVID-19 infection risks, prompting the use of online tools. Also, mental support needs increased as issues such as “loss of value” in their sports activities and connection with others became prevalent. Regular physical check-ups during and after the pandemic, proved to be valuable and emphasized the importance of planning for post-COVID retraining. Some issues were unique to NT; for example, six NT support staff reported difficulties in enhancing performance domestically due to a lack of competitive rivals, and three of them noted that training camps abroad were organized to avoid domestic quarantine and improve technical skills through competition with foreign athletes. Additionally, three staff emphasized the need for inter-organizational collaboration to secure large equipment. Another three staff organized the athletes' training in public park spaces and used playground equipment.

**Table 10:** Support staff responses [9 national team (NT) and 4 professional team (PT)] in interviews regarding the situation of athletes and support staff during the COVID-19 pandemic in general (including periods that cannot be specifically determined) (18 descriptions).

Categories	Subcategories	Descriptions	Total (NT, PT)
Level of restrictions	Social distancing and travel restrictions	Athletes had difficulty training due to infection control measures (e.g., training with masks).	2 (2, 0)
		A lack of domestic rivals made it difficult to improve performance in Japan.	6 (6, N)
		Foreign teams did not come because of strict Japanese restrictions related to the COVID-19 countermeasures.	1 (1, N)
Actions of athletes/staff	Communication	Information and Communication Technology (ICT) was utilized to promote communication.	2 (1, 1)
	Training	Training camps abroad were held to avoid domestic quarantine, improving technical skills through competition with foreign athletes.	3 (3, N)
		The team originally used a tracking system, so the support staff used that to set the training load.	2 (0, 2)
		Support staff used online tools to monitor training load and fatigue levels.	3 (2, 1)
		Since body composition was measured every month before the pandemic, it was possible to know the changes in body composition before and after isolation.	1 (0, 1)
		Due to the presence of several domestic rivals, there has been an improvement in their performance.	1 (1, 0)
		Balancing the risk of COVID-19 restrictions against the need for training and strengthening activities was difficult.	1 (1, 0)
		Athletes trained by utilizing public park spaces and playground equipment.	3 (3, 0)
		Athletes bought large training equipment (e.g., ergometers).	1 (0, 1)
	Recovery	Athletes bought recovery items.	2 (2, 0)
	Mental health	The team worked with a psychology specialist and set up a system where athletes could get counselling at any time.	1 (1, 0)
Challenges for athletes/staff	Challenges that happened	Some athletes had never had more than a week off before and did not know what to do.	1 (1, 0)
		Support staff were unable to quantify internal and external loads well.	2 (0, 2)
		Support staff were not able to work on site and monitor the players’ condition as they had been doing before.	1 (0, 1)
		There was a general trend of hesitancy towards training and sports activities.	2 (2, 0)

N, not applicable. Other minor descriptions are provided in Supplementary Table S5.

**Table 11:** Support staff responses [9 national team (NT) and 4 professional team (PT)] in interviews regarding achievements and challenges observed during the COVID-19 pandemic (34 descriptions).

Categories	Subcategories	Descriptions	Total (NT, PT)
Positive changes	Communication	Communication between support staff and athletes increased.	2 (2, 0)
		Online communication among athletes and support staff, as well as between athletes themselves, became more frequent.	3 (1, 2)
	Training	Support staff could evaluate athletes’ conditions online.	1 (0, 1)
		Athletes’ strengths were further enhanced.	1 (1, 0)
		Support staff developed more deliberate schedules.	1 (0, 1)
		Support staff maintained training durations even during the in-season period.	1 (0, 1)
		Support staff enhanced the variety of training programs.	1 (1, 0)
	Recovery	Careful monitoring and well-designed training programs led to a significant decrease in injuries compared to the past.	1 (1, 0)
	Others	Athletes’ autonomy increased.	1 (1, 0)
		Athletes’ individual attitudes towards the sport improved.	1 (1, 0)
		Athletes became more skilled at handling unexpected situations.	1 (1, 0)
Lessons learned	Training	Focusing on essential physiological elements in training proved effective.	1 (0, 1)
		Due to the insufficient training stimulus provided by bodyweight training for elite athletes, it was necessary to improve the home environment (i.e., purchasing small equipment, creating weights, and distributing them to the team).	3 (1, 2)
		Raising heart rate through bodyweight exercises was challenging.	1 (0, 1)
		Muscle strength could not be maintained without heavy weights.	1 (0, 1)
		Large equipment (e.g., stationary bikes, rowing machines) was effective for high-intensity training.	2 (2, 0)
		Yoga and breathing techniques were effective.	1 (0, 1)
		Investing in monitoring tools (e.g., heart rate monitors) was beneficial.	1 (0, 1)
		For quicker recovery, training intensity was more critical than volume, with high-intensity sessions recommended 2–3 times a week.	1 (1, 0)
		Proper recovery time was essential after breaks.	1 (0, 1)
	Mental health	Both support staff and athletes focused entirely on what they could achieve within their respective environments.	2 (2, 0)
Challenges and requests	Providing information	Enhanced information sharing was needed.	1 (1, 0)
	Training	Regular conditioning assessments and performance evaluations were prioritized.	3 (3, 0)
		Maintaining the operation of training facilities, such as national training centers, universities, and team facilities was considered essential during pandemics.	1 (1, 0)
		Sharing equipment among sports organizations was recommended, as individual organizations often lacked sufficient resources.	3 (3, N)
		Support staff's foundational knowledge of exercise physiology contributed to the development of standardized training principles.	1 (0, 1)
		Support staff developed adaptive training programs in response to the physical changes in athletes caused by training restrictions.	1 (1, 0)
	Mental health	A platform for athletes to connect with athletes from other sports was needed.	1 (1, 0)
		Mental health supports were recognized as essential.	1 (1, 0)
	Communication	Enhancing collaboration and information sharing among sports federations was vital, given the limited capacity of individual organizations.	1 (1, N)
	Validation	Encouraging in-depth discussions on balancing in-person training with COVID-19 infection risks was highly recommended.	1 (1, 0)
		Long-term effects on athletes unable to train due to cancellations or restrictions should be investigated.	1 (1, 0)
		Comparative evaluations of Japanese teams’ training programs with those of other countries should be conducted.	1 (1, 0)
	Others	Clear communication of government guidelines to local regions was critical, given variations in regional regulations.	1 (0, 1)

N, not applicable. Other minor descriptions are provided in Supplementary Table S6.

## 4 Discussion

This study explored the training practices of elite team and combat sport athletes during the COVID-19 pandemic, as reported by Japanese NT and PT support staff through semi-structured interviews. During the nationwide State of Emergency, most elite Japanese athletes received online training and training information from their team's support staff, which was deemed insufficient to achieve the desired training outcomes. After the nationwide State of Emergency, restrictions on training facility use remained in many areas in Japan, allowing only small-group training, and support staff were sometimes restricted from attending training sessions. Asymptomatic close-contact (non-infected) athletes were quarantined for two weeks, whereby they had access to large training equipment at their quarantine locations; however, some athletes experienced declines in physical fitness after this period. These findings suggest a degree of adaptability among elite athletes and support staff in managing training-related challenges during the early COVID-19 pandemic.

### 4.1 Training practices during the nationwide State of Emergency

In addition to previously discussed factors such as facility closures and travel restrictions (2, 4), the internal dynamics and organizational structure within teams were identified as additional factors negatively impacting training practices. Many Japanese NT athletes predominantly train with their respective affiliated teams, including university, company-sponsored, or club teams, and only assemble as a NT for training camps or international competitions. In these circumstances, support staff were often unable to provide guidance outside of these specific events (i.e., training camps or international competitions), resulting in many athletes largely managing their own training or relying on coaching from their affiliated teams. The environment surrounding NT athletes is therefore multi-layered, involving support staff from the national team, affiliated clubs, and personal coaching entities (18). Silva et al. (18) emphasized that effective communication among national, club, and personal staff is essential for enhancing players' performance and health, noting that responsibilities vary according to the specific period. Many NT support staff reported having no direct responsibilities for overseeing athletes' training, resulting in greater reliance on the support staff of their affiliated teams. These findings highlight the need for improved coordination and communication among all members of the athletes’ support network.

Many NT and PT support staff provided online training and training information, similar to other cases (3, 15, 16). They encouraged athletes to utilize the available training equipment to enhance training intensity. When existing equipment was deemed insufficient to achieve the desired training effects, they prescribed training programs tailored to the athletes' available resources, including equipment and environment. However, if the desired outcomes could not be achieved, they altered the training objectives and prescribed alternative exercises, such as increasing the number or speed of repetitions with lighter loads or incorporating bodyweight-based exercises. Nonetheless, commercially available small equipment often proved inadequate to provide the required training intensity for elite athletes, particularly for strength development or muscle hypertrophy. Additionally, training locations such as homes or hotels often lacked sufficient space, limiting dynamic training and extensive movements required by these athletes. Access to large equipment was available only to athletes from a limited number of NT and PT. Although outdoor training was permitted during the pandemic in Japan, elite athletes faced specific challenges, particularly voluntary restrictions imposed by the so-called “virus vigilantes”. Given the constraints, support staff had to design training plans that emphasized creativity and exercise variety. For instance, low-load training was adapted by adjusting the exercise volume, time under muscle tension, and training frequency to achieve hypertrophic outcomes comparable to traditional high-load training (19, 20). These approaches highlight the necessity of tailoring programs to the specific environment, ensuring that athletes can continue to make progress despite prevailing limitations.

Only two of the nine NT and PT support staff members reported that, due to having access to particular facilities, they were able to maintain sports-specific training intensity above 25% of pre-pandemic levels. This observation is considerably lower than the level reported in a previous study, where one-third of elite athletes were able to maintain their pre-lockdown training intensity (3). This discrepancy is likely attributed to the severe restrictions imposed on training during the pandemic, especially among team sports and combat sports, which require diverse physical demands and typically involve multiple participants training together in close proximity or through actual physical contact (3). Additionally, since the survey was conducted with NT and PT support staff, it is possible that the athletes' training prescribed by other support staff or self-managed by the athletes was not fully reflected or captured. In any case, the closure of dedicated training facilities for NT and PT had a pronounced impact on the ability of elite-level athletes in these sports. On the other hand, many NT and PT support staff reported that athletes were able to allocate more time than usual to cardiorespiratory and mobility exercises.

Conversely, there were positive aspects of the pandemic. Some athletes used this period to address long-standing injuries, undergoing surgical procedures and intensive rehabilitation that would have been challenging to accommodate during regular competitive seasons. Research has shown that a substantial proportion of elite athletes —up to 36%— experience chronic conditions that persistently affect their performance (21). Furthermore, by pausing intense training and competition, athletes were able to concentrate on treatments aimed at resolving these issues (22), potentially enhancing their overall physical health and longevity in sports. For some athletes, the lockdown proved to be a “blessing in disguise”, providing an “opportunity” to enhance some aspects of training and recovery that had previously been neglected (1).

Some support staff reported varying successes in maintaining athletes' motivation through online training. While some found virtual sessions effective, others encountered difficulties to keep athletes engaged over a prolonged period. This observation was particularly true for team and combat sports athletes accustomed to group training dynamics, as the absence of training partners was a critical factor contributing to decreased motivation (3). Consistent with previous studies, issues related to mental health and motivation were prevalent during the pandemic (23), highlighting the need for better access to mental health professionals or counselors (24). Reduced social interaction and collaborative training during the global pandemic also led to a deterioration in mental well-being among athletes (25, 26). While online meetings and training helped to some extent (e.g., motivation), athletes generally expressed a preference for training in environments with real partners. A supportive network of coaching staff, teammates, friends, and family (team environment) plays a vital role in maintaining emotional well-being and motivation (27). A study on Canadian national team athletes revealed that the pandemic caused significant disruptions in athletes' mental health, leading to low mood, anxiety, and stress, but also offered a rare opportunity for rest and recovery, as athletes with stronger mental performance skills coped better with challenges (24). These findings underline the need for integrated training programs that prioritize both physical and mental well-being, as well as recovery from chronic injuries, especially during prolonged isolation.

### 4.2 Training practices after the nationwide State of Emergency

Even after the nationwide State of Emergency, restrictions on training facility access persisted in different parts of the country. To minimize contact risk, training was often conducted in small groups, with limited access to support staff. In alignment with the return-to-training guidelines (28, 29), most NT and PT support staff implemented a gradual training progression to safely resume training activities. It's important that some studies reported maintenance of body mass, skinfold thickness, jump height among professional soccer players following a supervised remote-based physical training (30, 31). Among elite Japanese fencers, no changes in body composition were observed in males, while female fencers experienced a temporary increase in fat mass that returned to baseline within four months (32). In terms of “recovery” duration, studies on elite badminton players and combat sport athletes have shown that jump height, lower body strength, and body composition returned to pre-pandemic levels within eight weeks of retraining (33, 34). In a similar context, eight support staff (5 NT and 3 PT) perceived that endurance took longer to recover, typically two to four months. This perception of a more prolonged recovery for endurance capacity aligns with findings from professional soccer (35), where teams in leagues with shorter interruptions (e.g., German Bundesliga: 9-week match interruption, 3 weeks of group training cessation) maintained physical match performance, whereas those with prolonged disruptions (e.g., Spain's La Liga: up to 15-week match interruption, 8 weeks of group training cessation) showed pronounced performance declines. Although detraining effects generally become apparent after four or more weeks of training cessation (36), the present findings suggest that the nature and extent of training practices (including the potential effect on training supervision) during the State of Emergency were crucial in modulating these effects.

Regarding sports-specific skills, while objective assessments were not conducted in this study, some staff (3 NT and 1 PT) believed that the recovery would take even longer (i.e., over six months) due to severe restrictions on sports-specific training and the implementation of gradual, low-risk retraining protocols. This subjective view is consistent with observed changes in match characteristics and skill decrements in professional sports post-pandemic. For instance, studies noted declines in tennis service performance (37) and tactical shifts in soccer, such as increased conservatism and reduced attacking depth (38). These observations imply challenges in restoring optimal sports performance, particularly because highly specialized sports skills often require specific environmental cues, interactions with opponents, or team dynamics, all of which were severely constrained during the State of Emergency. Regardless of above, implementation of “bubble camps”, where athletes, coaches and support staff isolate together from society to continue regular training with full facility access (sport-specific practice, conditioning, recovery, etc.), emerged as a practical solution during lockdown situations (2, 39).

Almost half of the NT staff (4 out of 9) reported no unusual injuries, likely due to reduced training and less pressure to return to competition. In contrast, only one fourth of the PT staff reported no unusual injuries. Since professional leagues resumed within the same year, PT athletes were required to return to competition sooner, which led to injuries from inadequate pre-season preparation. For instance, the J.League (soccer) resumed on July 4, 2020, and a notable increase in muscle injuries was observed shortly after the State of Emergency (40). According to the study, the incidence reached 4.8 injuries per 1,000 h of exposure in May 2020, compared to 1.0 in May 2019. Conversely, B.league (basketball) resumed three months later, on October 2, and attested no notable increase in injuries, according to the league's official report (41). Furthermore, restrictions on international travel led to fewer opportunities for NT to train with world-class opponents and reduced international matches and training camps. To address these limitations, some NT organized training camps abroad to avoid domestic quarantine measures. One NT support staff member, however, expressed satisfaction with their training environment, attributing it to the local presence of strong rivals in Japan. Overall, following the nationwide State of Emergency, while general fitness was perceived to be restored within two months, the prolonged recovery of sports-specific performance emphasizes the necessity of balancing low-injury risk training protocols with comprehensive return-to-play plans (including management of the infection risks).

### 4.3 Training practices during and after two weeks quarantine

During the pandemic, athletes infected with COVID-19 were isolated in accordance with government guidelines and could not continue their training. Infected athletes may experience declines in performance capacity regardless of symptom status, though those exhibiting persistent, performance-impairing symptoms (such as cough, tachycardia, and fatigue) experience reduced ventilatory parameters and lower peak oxygen consumption values compared to those who remain symptom-free (42, 43). On the other hand, athletes quarantined due to close contact or other factors—particularly those without symptoms—were able to continue training within their quarantine settings. Among PT, all support staff reported securing large training equipment for athletes at their quarantine locations. This effort was likely driven by the financial resources of professional sports and the urgent need to resume competition within league schedules. However, many support staff noted declines in physical fitness following a two-week quarantine, though some observed a return to baseline within two weeks. Previous research has shown that a two-week break during a typical off-season increased skinfold thickness but maintained or even improved other fitness indicators among Australian football players, potentially due to recovery (44). However, these findings may not directly apply to the context of quarantine. Similarly, a study on university sprinters showed reductions in knee eccentric strength and fast concentric strength over a two-week off-season, while slow concentric strength and body composition remained unaffected (45). Fitness changes after a two-week quarantine may vary depending on physical activity levels. During the nationwide State of Emergency in Japan, physical activity levels clearly declined (46). A systematic review on bed rest indicated that endurance, neural function, and muscle strength can decline within just five days (47), suggesting substantial declines in physical performance are plausible after a two-week quarantine.

### 4.4 Limitation

This study used semi-structured interviews with support staff working with elite athletes, which may have introduced a recall bias. Furthermore, as this study relied on interviews with NT and PT support staff, training prescribed by other staff (e.g., from the athletes' clubs) or performed independently by athletes may not have been fully captured. Additionally, the subjective nature of evaluating training approaches (including quality, intensity, creativity, and innovation) may have been influenced by individual interpretation, potentially compromising precision.

## 5 Conclusion

This study highlights the diverse training approaches employed during the COVID-19 pandemic, as well as challenges faced by Japanese elite team sport and combat sport athletes due to restricted access to conventional training facilities and equipment. Online group and/or personal training sessions were considered valuable for maintaining fitness and motivation, although they were deemed inadequate for achieving optimal performance outcomes. The importance of mental health became evident as reduced social interaction, the absence of training partners, and uncertainty called for better mental health support that prioritizes athletes' overall well-being. Despite these difficulties, the pandemic also presented opportunities for some athletes to address chronic injuries, engage in thorough rehabilitation, explore alternative training methods (e.g., low-load exercises), and implement periodized return-to-play planning to preserve or regain physical aspects. These adaptive approaches, driven by the many initiatives of the support staff, were highly beneficial for the athletes. These findings emphasize the need for robust and flexible athlete-support systems capable of managing sudden uncertain situations and converting them into opportunities. Such systems should focus on alternative training approaches, communication networks, and holistic prioritization of athlete physical and mental health, and well-being through strong interdisciplinary collaboration to enhance preparedness and resilience for future challenges in high-performance contexts.

## Data Availability

The original contributions presented in the study are included in the article/Supplementary Material, further inquiries can be directed to the corresponding author.

## References

[1] Washif JA Hettinga FJ Ammar A Van Rensburg DCJ Materne O Trabelsi K Supporting athletes during a challenging situation: recommendations from a global insight of COVID-19 home-based training experience. BMC Sports Sci Med Rehabil. (2024) 16:83. 10.1186/s13102-024-00869-7 38622683 PMC11017558

[2] Washif JA Farooq A Krug I Pyne DB Verhagen E Taylor L Training during the COVID-19 lockdown: knowledge, beliefs, and practices of 12,526 athletes from 142 countries and six continents. Sports Med. (2022) 52:933–48. 10.1007/s40279-021-01573-z 34687439 PMC8536915

[3] Washif JA Sandbakk Ø Seiler S Haugen T Farooq A Quarrie K COVID-19 lockdown: a global study investigating the effect of athletes’ sport classification and sex on training practices. Int J Sports Physiol Perform. (2022) 17:1242–56. 10.1123/ijspp.2021-0543 35894967

[4] Zago M Lovecchio N Galli M. Players at home: physical activity and quality of life in 12–17 years-old football (soccer) players during the COVID-19 lockdown. Int J Sports Sci Coach. (2021) 17:626–36. 10.1177/17479541211041703 PMC9086221 35663129

[5] Rynne SB Mallett C Tinning R. High performance sport coaching: institutes of sport as sites for learning. Int J Sports Sci Coach. (2006) 1:223–34. 10.1260/174795406778604582

[6] Paludo A Karpinski K Silva S Kumstát M Sajdlová Z Milanovic Z. Effect of home training during the COVID-19 lockdown on physical performance and perceptual responses of team-sport athletes: a mini-review. Biol Sport. (2022) 39:1095–102. 10.5114/biolsport.2022.117040 36247967 PMC9536370

[7] Watanabe T Yabu T. Japan’s voluntary lockdown. PLoS One. (2021) 16:e0252468. 10.1371/journal.pone.0252468 34111163 PMC8191912

[8] Kitahara K Nishikawa Y Yokoyama H Kikuchi Y Sakoi M. An overview of the reclassification of COVID-19 of the infectious diseases control law in Japan. Glob Health Med. (2023) 5:70–4. 10.35772/ghm.2023.01023 37128229 PMC10130540

[9] Ministry of Health, Labour and Welfare. Response to COVID-19 After Classification Change [Online]. (2023). Available online at: https://www.mhlw.go.jp/stf/covid-19/kenkou-iryousoudan_00006.html (Accessed December 24, 2024)

[10] International Olympic Committee. Fifth coordination meeting for COVID-19 countermeasures at the Olympic and Paralympic Games Tokyo 2020 [Online]. (2020). Available online at: https://olympics.com/en/news/fifth-coordination-meeting-for-covid-19-countermeasures-at-tokyo-2020 (Accessed December 24, 2024)

[11] Jagim AR Luedke J Fitzpatrick A Winkelman G Erickson JL Askow AT The impact of COVID-19-related shutdown measures on the training habits and perceptions of athletes in the United States: a brief research report. Front Sports Act Living. (2020) 2:623068. 10.3389/fspor.2020.623068 33426521 PMC7785865

[12] Mon-López D García-Aliaga A Ginés Bartolomé A Muriarte Solana D. How has COVID-19 modified training and mood in professional and non-professional football players? Physiol Behav. (2020) 227:113148. 10.1016/j.physbeh.2020.113148 32858031 PMC7445487

[13] Pillay L Janse Van Rensburg DCC Jansen Van Rensburg A Ramagole DA Holtzhausen L Dijkstra HP Nowhere to hide: the significant impact of coronavirus disease 2019 (COVID-19) measures on elite and semi-elite South African athletes. J Sci Med Sport. (2020) 23:670–9. 10.1016/j.jsams.2020.05.016 32448749 PMC7235602

[14] Washif JA Kok LY James C Beaven CM Farooq A Pyne DB Athlete level, sport-type, and gender influences on training, mental health, and sleep during the early COVID-19 lockdown in Malaysia. Front Physiol. (2023) 13:1093965. 10.3389/fphys.2022.1093965 36714309 PMC9875133

[15] Schneider F Runer A Burkert F Aspang JSU Reider S Schneider H Digital workout versus team training: the impact of the COVID-19 pandemic on athletes. Sports Med Int Open. (2022) 6:E18–24. 10.1055/a-1734-5457 35462685 PMC9023314

[16] Taheri M Irandoust K Reynoso-Sánchez LF Muñoz-Helú H Cruz-Morales KN Torres-Ramírez R Effects of home confinement on physical activity, nutrition, and sleep quality during the COVID-19 outbreak in amateur and elite athletes. Front Nutr. (2023) 10:1143340. 10.3389/fnut.2023.1143340 37139442 PMC10150803

[17] Elo S Kyngäs H. The qualitative content analysis process. J Adv Nurs. (2008) 62:107–15. 10.1111/j.1365-2648.2007.04569.x 18352969

[18] Silva JR Buchheit M Hader K Sarmento H Afonso J. Building bridges instead of putting up walls: connecting the “teams” to improve soccer players’ support. Sports Med. (2023) 53:2309–20. 10.1007/s40279-023-01887-0 37480484 PMC10687197

[19] Schoenfeld BJ Grgic J Ogborn D Krieger JW. Strength and hypertrophy adaptations between low- vs. high-load resistance training: a systematic review and meta-analysis. J Strength Cond Res. (2017) 31:3508–23. 10.1519/JSC.0000000000002200 28834797

[20] Fyfe JJ Hamilton DL Daly RM. Minimal-dose resistance training for improving muscle mass, strength, and function: a narrative review of current evidence and practical considerations. Sports Med. (2022) 52:463–79. 10.1007/s40279-021-01605-8 34822137

[21] Clarsen B Rønsen O Myklebust G Flørenes TW Bahr R. The Oslo sports trauma research center questionnaire on health problems: a new approach to prospective monitoring of illness and injury in elite athletes. Br J Sports Med. (2014) 48:754–60. 10.1136/bjsports-2012-092087 23429267

[22] Patatas JM Winckler C. “We too will have to wait a year”: the impacts of COVID-19 and the postponement of the Tokyo 2020 paralympic games from Brazilian athletes and coaches’ perspectives. Sport Soc. (2022) 25:1252–72. 10.1080/17430437.2021.1997987

[23] Amos B Tibbert SJ. Exploration of the COVID-19 mental health implications in elite disabled athletes. J Sports Sci. (2024) 42:918–29. 10.1080/02640414.2024.2370151 38910346

[24] Dithurbide L Boudreault V Durand-Bush N Macleod L Gauthier V. The impact of the COVID-19 pandemic on Canadian national team athletes’ mental performance and mental health: the perspectives of mental performance consultants and mental health practitioners. Front Psychol. (2022) 13:937962. 10.3389/fpsyg.2022.937962 36059762 PMC9435585

[25] Carnevale Pellino V Lovecchio N Puci MV Marin L Gatti A Pirazzi A Effects of the lockdown period on the mental health of elite athletes during the COVID-19 pandemic: a narrative review. Sport Sci Health. (2022) 18:1187–99. 10.1007/s11332-022-00964-7 35693326 PMC9174028

[26] Andrew W Kristin H Kevin B Allison S Scott H Claudia R Impact of COVID-19 on the physical activity, quality of life and mental health of adolescent athletes: a 2-year evaluation of over 17000 athletes. Br J Sports Med. (2023) 57:359. 10.1136/bjsports-2022-105812 36424132

[27] Hussain T Wang D Li B. Psychological resilience in athletes during the COVID-19 pandemic: a qualitative insight. Acta Psychol. (2023) 240:104050. 10.1016/j.actpsy.2023.104050 37832493

[28] Caterisano A Decker D Snyder B Feigenbaum M Glass R House P CSCCa and NSCA joint consensus guidelines for transition periods: safe return to training following inactivity. Strength Cond J. (2019) 41:1–23. 10.1519/SSC.0000000000000477

[29] National Strength and Conditioning Association (NSCA) Covid-19 Return to Training Taskforce. COVID-19: NSCA Guidance on Safe Return to Training for Athletes. National Strength and Conditioning Association (NSCA). (2023). Available online at: https://www.nsca.com/contentassets/61c0fb0a476149848de009f1630fa457/nsca-covid-19-rtt.pdf (Accessed January 8, 2025)

[30] Cohen DD Restrepo A Richter C Harry JR Franchi MV Restrepo C Detraining of specific neuromuscular qualities in elite footballers during COVID-19 quarantine. Sci Med Footb. (2021) 5:26–31. 10.1080/24733938.2020.1834123 35077322

[31] Anderson L Flannigan C Polychronopoulos P Mackenzie R Drust B Milsom J. Lessons from the COVID-19 pandemic: insights into effective training strategies for physical development in football. Int J Sports Sci Coach. (2023) 18:403–13. 10.1177/17479541221081782 38603107 PMC9014339

[32] Yasuda J Kondo E Takai E Eda N Azuma Y Motonaga K The effects of the COVID-19 environments on changes in body composition in Japanese elite fencing athlete. Sports. (2021) 9:95. 10.3390/sports9070095 34202335 PMC8309863

[33] Valenzuela PL Rivas F Sánchez-Martínez G. Effects of COVID-19 lockdown and a subsequent retraining period on elite Athletes’ workload, performance, and autonomic responses: a case series. Int J Sports Physiol Perform. (2021) 16:1707–11. 10.1123/ijspp.2020-0735 33873155

[34] Tan E Montalvo S Gonzalez MP Dietze-Hermosa M Min S Dorgo S. Changes in vertical jump performance and body composition before and after COVID-19 lockdown. J Hum Sport Exer. (2023) 18:224–41. 10.14198/jhse.2023.181.18

[35] Thron M Düking P Härtel S Woll A Altmann S. Differences in physical match performance and injury occurrence before and after the COVID-19 break in professional European soccer leagues: a systematic review. Sports Med - Open. (2022) 8:121. 10.1186/s40798-022-00505-z 36178557 PMC9523642

[36] Mujika I Padilla S. Detraining: loss of training-induced physiological and performance adaptations. Part II. Sports Med. (2000) 30:145–54. 10.2165/00007256-200030030-00001 10999420

[37] Ishihara T Robin N Naito T Murata M Crespo M. Effects of the COVID-19 pandemic on professional tennis players’ match statistics: a large-scale population-based study. Scand J Med Sci Sports. (2022) 32:1516–8. 10.1111/sms.14216 36097685

[38] Fernández-Cortés J Gómez-Carmona CD Mancha-Triguero D García-Rubio J Ibáñez SJ. Effect of COVID-19 on key performance indicators of Spanish professional soccer league. J Funct Morphol Kinesiol. (2024) 9:35. 10.3390/jfmk9010035 38535415 PMC10970726

[39] Washif JA Ammar A Trabelsi K Chamari K Chong CSM Mohd Kassim SFA Regression analysis of perceived stress among elite athletes from changes in diet, routine and well-being: effects of the COVID-19 lockdown and “bubble” training camps. Int J Environ Res Public Health. (2022) 19:402. 10.3390/ijerph19010402 PMC8744934 35010662

[40] Matsunaga R Nagao M Akagi R Fukai A Sando T Ikeda H Impact of the COVID-19 pandemic on injury incidence in Japanese male professional soccer players. Orthop J Sports Med. (2023) 11:23259671221149373. 10.1177/23259671221149373 36860773 PMC9969458

[41] Japan_Professional_Basketball_League. B.LEAGUE 2022-23 Season Injury Report (in Japanese) [Online]. (2023). Available online at: https://www.bleague.jp/files/user/Injury%20Report.pdf (Accessed January 8, 2025).

[42] Wilson MG Hull JH Rogers J Pollock N Dodd M Haines J Cardiorespiratory considerations for return-to-play in elite athletes after COVID-19 infection: a practical guide for sport and exercise medicine physicians. Br J Sports Med. (2020) 54:1157–61. 10.1136/bjsports-2020-102710 32878870 PMC7513247

[43] Vollrath S Bizjak DA Zorn J Matits L Jerg A Munk M Recovery of performance and persistent symptoms in athletes after COVID-19. PLoS One. (2022) 17:e0277984. 10.1371/journal.pone.0277984 36477204 PMC9728914

[44] Buchheit M Morgan W Wallace J Bode M Poulos N. Physiological, psychometric, and performance effects of the christmas break in Australian football. Int J Sports Physiol Perform. (2015) 10:120–3. 10.1123/ijspp.2014-0082 24806508

[45] Yamashita D Hirata K Yamazaki K Mujika I Miyamoto N. Effect of two weeks of training cessation on concentric and eccentric knee muscle strength in highly trained sprinters. PLoS One. (2023) 18:e0288344. 10.1371/journal.pone.0288344 37418449 PMC10328257

[46] Yamada Y Namba H Date H Kitayama S Nakayama Y Kimura M Regional difference in the impact of COVID-19 pandemic on domain-specific physical activity, sedentary behavior, sleeping time, and step count: web-based cross-sectional nationwide survey and accelerometer-based observational study. JMIR Public Health Surveill. (2023) 9:e39992. 10.2196/39992 36634262 PMC9953987

[47] Spiering BA Weakley J Mujika I. Effects of bed rest on physical performance in athletes: a systematic and narrative review. Sports Med. (2023) 53:2135–46. 10.1007/s40279-023-01889-y 37495758 PMC10587175

