# Supplementary material for: Training practices of Japanese elite team and combat sport athletes during the COVID-19 pandemic: an interview study with support staff
Source: Front Sports Act Living. 2025 Sep 4;7:1557774. doi: 10.3389/fspor.2025.1557774 (PMC12443745; doi:10.3389/fspor.2025.1557774)
Supplement: Supplementary file 1 [file Table1.docx]

**Supplemental materials**

**Table S1.** Support staff responses (8 National Team [NT] and 4 Professional Team [PT]) in interviews regarding the situation of athletes and support staff during the nationwide State of Emergency (7 descriptions).

| Categories | Subcategories | Descriptions | Total  (NT, PT) |
| --- | --- | --- | --- |
| Level of restrictions | Facility closure/ restriction | Athletes in rural areas could use commercial gyms, while those in urban areas had no such access. | 1 (1, 0) |
|  | Self-isolation | Support staff advised athletes to be cautious of surroundings due to “virus vigilantes.” | 1 (0, 1) |
| Actions of athletes/staff | Communication | Support staff explained home training to athletes' families to gain their understanding. | 1 (1, 0) |
|  | Training | Support staff prescribed increased training frequency to maintain training volume due to limited facility access. | 1 (0, 1) |
|  |  | Support staff incorporated injury prevention exercises into the training program. | 1 (1, 0) |
| Challenges for athletes/staff | Training | Athletes could not access facilities due to time restrictions. | 1 (0, 1) |
|  |  | Athletes with jobs had difficulty finding time to train due to increased work commitments. | 1 (1, 0) |

**Table S2.** Support staff responses (9 National Team [NT] and 4 Professional Team [PT]) in interviews regarding the situation of athletes and support staff after the nationwide State of Emergency (6 descriptions).

| Categories | Subcategories | Descriptions | Total  (NT, PT) |
| --- | --- | --- | --- |
| Actions of athletes/support staff | Training | Athletes resumed full-team practice in July to align with the start of the regular season. | 2 (0, 2) |
|  |  | Support staff received athletes' heart rate data remotely. | 1 (0, 1) |
|  |  | Support staff prescribed endurance training with varied intensities, along with diverse training options. | 1 (1, 0) |
| Challenges for athletes/staff | Recovery | Restrictions on medical staff interactions created confusion, but adaptation was quick. | 1 (1, 0) |
| Unusual injury | Muscle | Quadriceps injuries occurred. | 1 (0, 1) |
|  | Chronic issues | Existing chronic conditions remained unchanged. | 1 (1, 0) |

**Table S3.** Support staff responses (9 National Team [NT] and 4 Professional Team [PT]) in interviews regarding the situation of athletes and support staff during a two-week quarantine. (13 descriptions).

| Categories | Subcategories | Descriptions | Total  (NT, PT) |
| --- | --- | --- | --- |
| General quarantine |  |  |  |
| Actions | Recovery | Support staff prescribed stretching exercises for athletes during close contact isolation. | 1 (0, 1) |
|  | Providing information | Support staff advised athletes to maintain immune health during close contact isolation. | 1 (1, 0) |
| Challenges for athletes/staff | Training | Facility access arrangements during quarantine were inadequate, leading to home isolation. | 1 (1, 0) |
|  |  | Support staff prescribed endurance training with varied intensities (e.g., different step lengths and frequencies). | 1 (1, 0) |
| Group quarantine (including the Athlete Track) | | |  |
| Challenges for athletes/staff | Social distancing and travel restrictions | Athletes trained on the track, but practice partners were limited. | 1 (1, 0) |
| Quarantine from international competitions | | |  |
| Actions of athletes/staff | Advance preparation | The support staff did not have athletes prepare training equipment in advance to keep their focus on competition. | 1 (1, 0) |
|  |  | Support staff instructed athletes to prepare small equipment (e.g., tubes, slings) before leaving Japan. | 1 (1, 0) |
| Quarantine from Overseas (two weeks) | | |  |
| Actions of athletes/staff | Training | Support staff conducted in-person training sessions for athletes under government-approved conditions. | 1 (0, 1) |
|  |  | Athletes trained online with personally contracted trainers. | 1 (0, 1) |
|  | Communication | Teams communicated with newly contracted foreign athletes to ensure that their goals were aligned. | 1 (N, 1) |
| Challenges for athletes/staff | Communication | Newly contracted foreign athletes struggled with insufficient pre-arrival communication and could not bring equipment to the hotel. | 1 (N, 1) |
|  | Others | Newly contracted foreign athletes faced difficulties entering Japan due to various regulations. | 1 (N, 1) |
|  |  | Athletes' families faced difficulties in entering Japan. | 1 (N, 1) |

Note: N, not applicable.

**Table S4.** Support staff responses (9 National Team [NT] and 4 Professional Team [PT]) in interviews regarding the situation of athletes and support staff after a two-week quarantine (4 descriptions).

| Categories | Subcategories | Descriptions | Total  (NT, PT) |
| --- | --- | --- | --- |
| Challenges for athletes/staff | Others | Under league regulations, matches were canceled after a positive case but rescheduled within two days. | 1 (N, 1) |
|  |  | Quarantined athletes were compelled to participate in a national team selection despite not being in peak condition. | 1 (1, N) |
| Unusual injury | Muscle | Athletes experienced intense workouts driven by excitement and anxiety, leading to muscle strain-like symptoms. | 1 (1, 0) |
| Change in performance | - | College athletes faced training disruptions due to academic commitments, resulting in unexpected drops in physical strength. | 1 (1, N) |

Note: N, not applicable.

**Table S5.** Support staff responses (9 National Team [NT] and 4 Professional Team [PT]) in interviews regarding the situation of athletes and support staff during the COVID-19 pandemic in general (including periods that cannot be specifically determined) (11 descriptions).

| Categories | Subcategories | Descriptions | Total  (NT, PT) |
| --- | --- | --- | --- |
| Level of restrictions | Social distancing and travel restrictions | Newly contracted foreign athletes were unable to maintain their fitness levels before joining the Japanese team because restrictions on training were more severe in their home countries compared to Japan | 1 (N, 1) |
| Actions of support staff | Provision of information | Support staff provided exercises that athletes could perform with their children at home. | 1 (1, 0) |
| Actions of athletes/staff | Training | Training was more manageable abroad than in Japan due to less strict restrictions. | 1 (1, 0) |
|  | Communication | Support staff explained infection prevention measures abroad to gain understanding from the national federations. | 1 (1, 0) |
| Challenges for athletes/staff | Training | Complicated facility access procedures caused hesitation among athletes and support staff. | 1 (1, 0) |
|  | Others | Managing cases of infection abroad was challenging (hospital coordination, meal provision, and health management). | 1 (1, 0) |
|  |  | Athletes and support staff became infected with COVID-19 during overseas training camps. | 1 (1, 0) |
|  |  | English-speaking staff faced additional burdens managing cases of COVID-19 infection during overseas training camps. | 1 (1, 0) |
|  |  | Regional (prefectural) variations in isolation rules by the local public health centers created unfair conditions for professional teams. | 1 (N, 1) |
|  |  | Activity restrictions for close contact rules varied across regions (prefectures) and time periods, limiting training opportunities. | 1 (0, 1) |
|  |  | Strict criteria resulted in all athletes being classified as close contacts due to traveling together on a chartered bus. | 1 (0, 1) |

Note: N, not applicable.

**Table S6.** Support staff responses (9 National Team [NT] and 4 Professional Team [PT]) in interviews regarding achievements and challenges observed during the COVID-19 pandemic (5 descriptions).

| Categories | Subcategories | Descriptions | Total  (NT, PT) |
| --- | --- | --- | --- |
| Way of thinking | Mental health | Athletes and staff did not perceive the situation as urgent, believing that the situation was consistent worldwide. | 1 (1, 0) |
|  |  | Athletes and staff did not perceive the situation as urgent, believing that the situation was consistent across Japan. | 1 (1, 0) |
|  |  | Athletes experienced anxiety when competing against international opponents who had been able to continue training. | 1 (1, 0) |
| Challenges and requests | Training | The availability of team-exclusive facilities for training and sport-specific practice was highly desired. | 1 (1, N) |
| Positive changes | Others | Team activities continued without any reported cases of COVID-19 infection (as of the interview). | 1 (1, 0) |

Note: N, not applicable.
